# Endometrial polypectomy using tissue removal device or electrosurgical snare: a randomised controlled trial

**DOI:** 10.52054/FVVO.14.3.035

**Published:** 2022-09-30

**Authors:** J van Gemert, M.C. Herman, P Beelen, P.M. Geomini, M.Y. Bongers

**Affiliations:** Department of Obstetrics and Gynecology, Máxima MC, De run 4600, 5504 DB Veldhoven, the Netherlands; Department of General Practice, University of Maastricht, P. Debyeplein 1, 6229 HA Maastricht, The Netherlands; Department of Obstetrics and Gynecology, GROW School for Oncology and Developmental Biology, Maastricht University Medical Center, P. Debyelaan 25, 6229 HX Maastricht, The Netherlands; Department of Obstetrics and Gynecology, Jeroen Bosch ziekenhuis, Henri Dunantstraat 1, 5223 GZ ‘s-Hertogenbosch, The Netherlands

**Keywords:** endometrial polyps, endometrial polypectomy, outpatient care, electrosurgical polyp snare, tissue removal device

## Abstract

**Background:**

Hysteroscopic transcervical resection of endometrial polyps is a widely used method and is increasingly performed in office or outpatient care. To ensure patient comfort is key, smaller instruments are preferred while also achieving a complete resection of the pathology.

**Objectives:**

To evaluate the effectiveness of the electrosurgical polyp snare (DPS) in comparison with a tissue removal device (TRD).

**Materials and Methods:**

This was a randomised controlled non-inferiority trial which included 66 women with symptomatic endometrial polyps who had been referred to the gynaecological outpatient clinic at the Máxima Medical Centre, Veldhoven, the Netherlands. The patients were randomly allocated by using sealed envelopes to treatment with either the DPS (Duckbill®, Cook) or the TRD (Truclear™, Medtronic). Clinicians and patients were not blinded to the treatment performed. An independent observer blindly assessed the results.

**Main outcome measures:**

The primary outcome was the complete removal of the endometrial polyp. Secondary outcomes were woman acceptability and pain during the procedure, operating time, peri-operative and immediate postoperative complications.

**Results:**

57% of the polyps in the DPS group and 95% in the TRD group were completely removed (risk difference -0.39; 95% CI: -0.60 to -0.15). Average operating time was longer with DPS compared to the TRD (11.7 min. vs. 6.8 min., p = 0.018). The number of insertions of the hysteroscope was higher with the DPS compared to the TRD (3.9 vs. 1.7, p <0.001). One serious adverse event, a uterine perforation, occurred in the DPS group.

**Conclusion:**

The TRD was superior to the DPS in completeness of polyp removal.

**What is new?:**

TRD has a higher rate of complete polyp resection, overall safety and higher patient satisfaction, this instrument should be considered as the preferred option in outpatient and office gynaecology.

## Introduction

Endometrial polyps are common growths in the inner lining of the uterus. They occur in both pre- and post- menopausal women and are often asymptomatic (Dreisler et al., 2009; Lieng et al., 2009). The most common symptom is abnormal uterine bleeding (Dreisler et al., 2009; Lieng et al., 2010). The majority of polyps are benign, yet in the post-menopausal population, 4-6% of polyps are due to pre-malignant or malignant changes, with 1-2% in premenopausal patients due to pre- malignant or malignant changes (Lee et al., 2010; Van Hanegem et al., 2017). Histopathologic examination of the polyp is necessary to confirm if it is benign and if suspicious changes are confirmed, it is essential to ensure complete removal as if polyp tissue remains in-utero, there is a chance of evolution to endometrial cancer (Lieng et al., 2010).

The first-choice treatment for symptomatic endometrial polyps is hysteroscopic removal. With advancement of technique, hysteroscopic surgery is now mainly taking place in the outpatient setting with reduced hysteroscope diameter and ancillary instrumentation combined with enhanced visualisation, mostly without the need for local anaesthesia (Lieng et al., 2010; American Association of Gynecologic Laparoscopists, 2012; Diwakar et al., 2015).

Several hysteroscopic techniques exist to remove endometrial polyps (Noventa et al., 2015), such as the use of an electrosurgical snare and the use of a hysteroscopic tissue removal device (TRD). The Duckbill^®^ Polyp snare (DPS) distributed by Cook Medical, a monopolar electrosurgical snare, was first been described in 2005 (Timmermans and Veersema, 2005). Studies on polyp removal with the use of the electrosurgical snare have shown it is an efficient and safe method that is well tolerated by patients (Timmermans and Veersema, 2005). These benefits are also combined with a relatively low purchase price of 55 Euro. However, the technique requires a high level of skill and experience in outpatient hysteroscopic surgery, which has been resulted in a limited adoption of polyp snare procedures.

Hysteroscopic tissue removal became available as a treatment option for endometrial polyps in 2005. The TruClear™ hysteroscopic tissue removal device (Medtronic) has the ability to both cut and retrieve the polyps at the same time. The fact that the polyp tissue fragments are subsequently sucked through the tissue removal device, reduces the risk of perforation, which is more likely with multiple insertions of the resectoscope (Munro, 2010). Two recently conducted randomised controlled trials showed an overall efficacy of complete resection of 92-98% (Smith et al., 2014; Pampalona et al., 2015). This device comes with increased cost as the tissue removal device system requires acquisition of a specific hysteroscope which will create additional costs. The purchase price of the TruClear™ hysteroscopic tissue removal device is 304,32 Euro, not including the hysteroscope.

Both methods have very specific properties and are widely used in gynaecological practice. Nevertheless, hysteroscopic tissue removal is more widely used for polyp resection compared to hysteroscopic snare procedures because of the ease in use. There is no agreed first-choice method or gold standard for the removal of endometrial polyps as studies on both effectiveness, safety and costs are limited.

Currently several disposables, but also reusable (for example Bigatti Shaver, Storz) tissue removal devices are available. The aim of this study is to evaluate the effectiveness of the electrosurgical polyp snare in comparison with the TruClear hysteroscopic tissue removal device.

## Methods

### Population

Women with symptomatic endometrial polyps, over the age of 18 and were fluent in Dutch were eligible to be included in this study. They had been referred to the gynaecological one stop clinic at the Máxima Medical Centre by their General Practitioner if they had complaints of heavy menstrual bleeding, intermenstrual bleeding, or postmenopausal bleeding. A transvaginal ultrasound and/or saline infusion sonohysterography (SIS) was performed and confirmed the presence of an endometrial polyp. The majority of polypectomies were performed immediately at the clinic, yet if the patient preferred to undergo sedation, or the intervention was too uncomfortable, the procedure was scheduled to occur at another stage under sedation.

Patients were excluded from the trial if there was a suspicion of malignancy or if written informed consent was not obtained or withdrawn at any stage. The number and size of polyps were not exclusion criteria.

### Randomisation and Blinding

Patients were randomised in a 1:1 ratio using sealed, numbered envelopes. An independent researcher concealed 220 envelopes with either treatment by the electrosurgical snare or the hysteroscopic tissue removal device. One envelope was opened just before the intervention to determine which equipment was used. Blinding of both women and clinicians was not possible because of the use of different equipment. Results of the procedures were blindly assessed by independent observers.

### Study procedures and co-interventions

Women were treated by four experienced gynaecologists. Hysteroscopy was performed either in the outpatient clinic or in the operating room under sedation, depending on the woman’s preference. Level 1 of pain management was used in outpatient care (Carugno et al., 2021). No specific sedative medication was recommended prior to the procedure in the outpatient clinic. All women were advised to use paracetamol 1000mg and naproxen 500mg as analgesia one hour before the procedure. If oral non-steroidal anti-inflammatory drugs were contraindicated, only paracetamol 1000mg was recommended. Level 3B of pain management was used for women treated in the operating room (Carugno et al., 2021). IV propofol was used for conscious sedation and administered by a sedationist. The model of care was outpatient care, yet if sedation were used, ambulatory care was provided. If complications occurred which required prolonged hospital stay, inpatient care was recommended.

Women were randomised to either de Duckbill® polyp snare (Cook Medical) or the Truclear™ hysteroscopic tissue removal device (Medtronic). The Duckbill® polyp snare can be introduced through the working channel of 2.8mm hysteroscope as a minimum to ensure vision of the operating field. It operates by placing an electrosurgical loop to the base of the polyp after which the loop is closed, and the wire is charged with monopolar current, and the polyp is removed from the endometrial surface. Then the floating polyp can be caught with the snare and removed. Use of hysteroscopic grasping forceps introduced through the working channel after removing the snare was also used to collect and remove the polyp from the uterine cavity. Use of hysteroscopic scissors was noted as co-intervention and included in the judgement of completeness of resection.

The Truclear™ hysteroscopic tissue removal device is a system that incorporates a disposable mechanical cutting device, with a shaver blade on the outside and suction tube on the inside. Both the outer sheath and inner tube have corresponding apertures for simultaneous cutting and suction of polyp tissue. The suction tube creates negative pressure to absorb the tissue near the apertures. The shaver blade can penetrate the polyp tissue with ease and prevent ejection of tissue from the aperture during closure. The speed of the shaver blade is calculated to leave enough time for tissue fragments to enter. The blade is connected to a vacuum device which aspirates the tissue through a side-facing aperture in the outer sheath. Distension fluid and resected polyp tissue are transferred to a tissue trap and vacuum canister through a tube protruding from the proximal end of the hand piece connected to the blade (Franchini et al., 2021).

It only works with the accompanying hysteroscope with an outer diameter of 5mm and a 2.9mm tissue removal device. If hysteroscopic grasping forceps or other devices needed to be used to retrieve the polyp, this was noted as a co-intervention.

### Outcome measures and follow-up

The primary outcome was defined as successful resection of the endometrial polyp. The outcome was confirmed by making a final hysteroscopic picture after treatment with either method. The operating gynaecologist scored the removal as either ‘complete’ or ‘incomplete’, which means full detachment from the endometrium and complete removal from the endometrial cavity. This picture was then linked to the study serial number and reviewed by a second, blinded, independent gynaecologist, who also marked it as ‘complete’ or ‘incomplete’ removal. If no consensus between the two gynaecologists were obtained, the judgement of the researcher, who was not involved in the treatment procedure and was also blinded for treatment technique, resulted in the final decision.

Secondary outcomes included the visual analogue scale (VAS) score during the resection of the polyp and overall, VAS score. This was noted by the nurse on the case record form (CRF) (0 for no pain, 100 for worst imaginable). This was only possible in the outpatient clinic, where no sedation was used (n=43). General acceptability of the procedure on an ordered Likert scale (fully acceptable, moderately acceptable, unacceptable, or highly unacceptable) was also obtained. Operating time was measured in minutes.

Data on serious- and adverse event were collected during the study follow-up. Adverse events were defined as any undesirable experience occurring during the study. Perioperative complications or failed procedures were noted on the CRF form by the gynaecologist which included poor sight during the procedure, non-tolerability of the procedure by the patient and occurrence of a vasovagal collapse. Postoperative complications and evaluation of bleeding or pain were scored two weeks after treatment by a telephone appointment (e.g., fever/ infection, post-treatment abnormal or prolonged vaginal bleeding or ongoing pain).

### Study oversight

An independent trial steering committee and an independent medical ethics committee, the METC of Maxima MC, provided oversight of the study. Trial registration: NL 56432.015.16.

### Statistical methods

The effectiveness of the electrosurgical snare relative to the hysteroscopic tissue removal device was expressed in terms of risk difference in completeness of resection, using 95% confidence intervals (CI) to express statistical uncertainty. Statistical analysis was performed according to the intention-to-treat principle with the hypothesis that the electrosurgical snare would be non-inferior to the hysteroscopic tissue removal device. The electrosurgical snare is advantageous due to lower costs when compared to the tissue removal device and therefore preferred in case non-inferiority is proven. A non-inferiority trial was chosen to show that the treatments are equally effective.

Previous studies have shown a mean overall efficacy of 95% for the hysteroscopic tissue removal device (Pampalona et al., 2015; Hamerlynck et al., 2015). Assuming 5% incomplete resections in both arms, a limit for non-inferiority of 10% was chosen.

Non-inferiority of the electrosurgical snare would be shown if the lower limit of the 95% CI for the risk difference in completeness of resection did not exceed the predetermined non-inferiority margin of 10%, with a corresponding p-value of <0.05.

### Sample size

With a power of 80% (p=0.05) and a 10% drop out 220 participants were required in total, which means 110 participants per treatment arm.

### Data analysis

Statistical software SPSS was used for all statistical analysis. All statistical analysis was performed according to the intention-to-treat principle, which included cross-over. Primary outcome was also performed by per protocol analysis, which included only those women who received their allocated treatment.

For the primary outcome, completeness of polyp removal, point estimates and two-sided 95% confidence intervals (CI) were calculated for the risk difference; this study could only declare non- inferiority if the lower band of the confidence interval did not exceed the 10% margin. Independent samples T test and X2 tests were used to assess statistical significance. Logistic regression was used for subgroup analysis for primary outcome.

## Results

### Women and randomisation

In total, 81 women were eligible for this study between November 2016 and December 2018. The study was ceased prematurely in December 2018, because the polyp snare was withdrawn from the market by the industry due to reported complications. In some cases, the snare loop did not completely retract and/or deformation of the snare loop occurred. It has been reported that this could led to uterine perforation. We reported the METC of this premature discontinuation of the trial.

**Figure 1:**
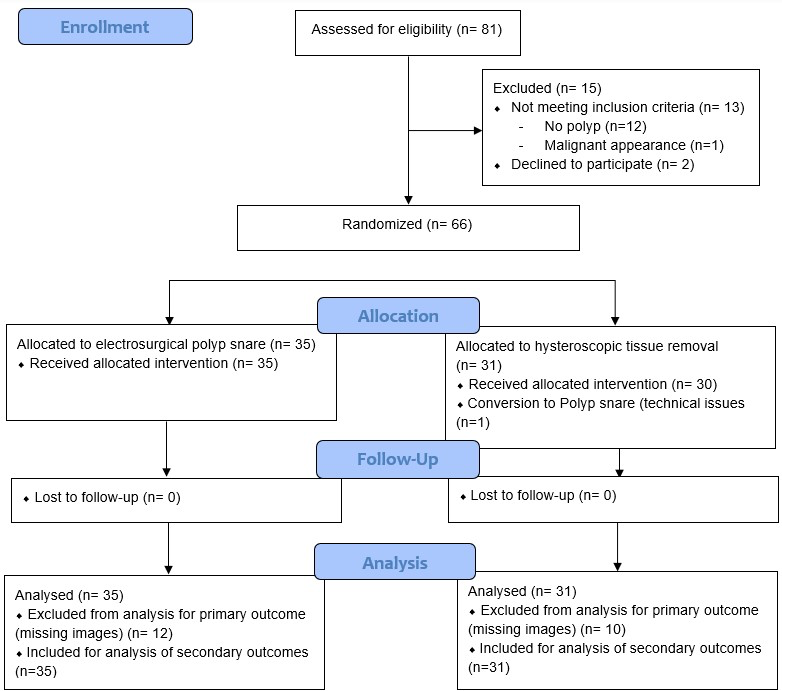
Flow chart showing enrolment, randomisation and follow up of participants.

Of the 81 women eligible for this study, fifteen women were excluded for randomisation. N=13 women because of not meeting the inclusion criteria, and two women withdrew informed consent. A total of 66 women were randomised: 31 in the hysteroscopic tissue removal device group and 35 in the electrosurgical snare group (Figure 1). Baseline characteristics of the women were similar in both groups (Table I).

**Table I:** Baseline characteristics of women undergoing hysteroscopic polypectomy. Values are numbers (percentages) unless stated otherwise.

	Electrosurgical snare (N=35)	Hysteroscopic tissue removal device (N=31)
Mean age (years) (SD)	59.7 (10.0)	56.2 (14.5)
Mean parity(SD)	2.0 (1.3)	1.8 (1.1)
Previous uterine interventions	9 (25.7)	8 (25.8)
Mean endometrial thickness (mm) (SD)	9.4 (4.3)	8.9 (5.6)
Postmenopausal	27 (77.1)	20 (64.5)

Unfortunately, the images of the first 22 women included in the study were deleted due to a technical fault in the computer system which deleted all hysteroscopic images. The images could not be recovered by members of the medical IT staff. Therefore, this data could not be included for the primary outcome, i.e., completeness of resection. In 44/66 (67%) of the included women the hysteroscopic images could be used for analysis of the primary outcome measure. Images were present of 21/31 (68%) women in the hysteroscopic tissue removal device group and 23/35 (66%) in the electrosurgical snare group. Baseline characteristics of women in the missing images group and images present group were similar (Table II).

**Table II:** Baseline characteristics of women of which the images are present versus women with missing images. Values are numbers (percentages) unless stated otherwise.

	Images present (N= 44)	Missing images (N= 22)
Mean age (years) (SD)	56.0 (12.2)	62.1 (11.8)
Mean parity (SD)	2.0 (1.3)	1.8 (1.0)
Previous uterine interventions	12 (27.3)	5 (22.7)
Mean endometrial thickness (mm) (SD)	8.4 (4.4)	10.7 (5.6)
Postmenopausal	28 (63.6)	19 (86.4)

### Primary outcome: completeness of resection

Consensus between the two gynaecologists about completeness of resection was obtained in 40/44 (91%) of the cases. In the other cases, the judgement of the blinded researcher resulted in the final decision.

**Figure 2:**
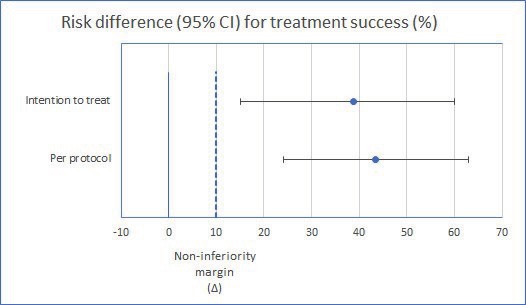
Risk differences (%) and 95% confidence intervals comparing treatment successs between the electrosurgical snare and hysteroscopic tissue removal device. Blue dashed line at 10% indicates non-inferiority margin.

Overall, 57% (13/23) of the women in the electrosurgical snare group and 95% (20/21) in the hysteroscopic tissue removal group had a complete resection of the polyp (p= 0.003). The risk difference between the electrosurgical snare and the hysteroscopic tissue removal device is 39% (0.39; 95% CI: 0.60 to 0.15). Per protocol analyses showed a risk difference of 44% (0.44; 95% CI: 0.63 to 0.24). The confidence intervals showed that risk difference was not within the 10% margin of non-inferiority, making the electrosurgical snare inferior compared to the hysteroscopic tissue removal device (Figure 2). Logistic regression analysis showed no significant differences in completeness of polyp removal corrected for baseline characteristics.

Reasons for incomplete resection by operating gynaecologist were poor visibility, technical difficulties, patient reported pain during procedure, size and/or location of polyp. Incomplete resected polyps using the electrosurgical snare were mainly located fundal (n=4) and in the uterine horns (n=3), or sessile polyps (n= 5). The most common site of an incomplete resected polyp with the hysteroscopic tissue removal device was in the posterior wall.

### Secondary outcomes

All 66 randomised women were included in the analyses of secondary outcome measures.

**Table III:** Secondary outcome measures of all 66 women. Values are numbers (percentages) unless stated otherwise.

	Electrosurgical snare (N=35)	Hysteroscopic tissue removal device (N=31)	P-value
Mean operating time in minutes (SD)	11.7 (9.3)	6.8 (5.7)	0.018
Mean number of insertions of hysteroscope (SD)	3.9 (2.5)	1.7 (0.9)	<0.001
Per-operative complications	1 (2.9)	0	0.173
Postoperative complaints	8 (22.9)	11 (35.5)	0.131

### Pathophysiology

Histopathological examination of the resected polyps showed that 64/66 (96%) were benign polyps. In one of the endometrial polyp atypia was found, and one polyp showed a malignancy. Both polyps were resected completely and both women received additional treatment by hysterectomy and oophorectomy. None of the incomplete resected polyps were premalignant.

### Patient acceptability and pain during the procedure

There were no significant differences in pain scores in either the electrosurgical snare group or hysteroscopic tissue removal group (Table IV). In the electrosurgical snare group 15/22 (68%) patients found the procedure fully acceptable.

**Table IV:** Use of analgesics, VAS and acceptability of the 43 women undergoing the procedure in the outpatient clinic. Values are numbers (percentages) unless stated otherwise.

	Electrosurgical snare (N=22)	Hysteroscopic tissue removal device (N=21)	P-value
Use of oral analgesics prior to the procedure	9 (40.9)	9 (42.8)	0.449
Mean VAS during resection/tissue removal (SD)	31.0 (20.0)	30.6 (23.9)	0.956
Mean overall VAS (SD)	31.5 (21.7)	39.3 (21.1)	0.284
Acceptability			
Completely acceptable	15 (68.2)	12 (57.1)	0.229
Reasonably acceptable	7 (31.8)	7 (33.3)	0.458
Unacceptable	0	2 (9.5)	0.072
Very unacceptable	0	0	

In the hysteroscopic tissue removal group 12/21 (57%) patients scored a fully acceptable procedure. There were no scored in the electrosurgical snare group for the procedure as unacceptable or very unacceptable. In the hysteroscopic tissue removal device group 2/21 (9.5%) scored the procedure as unacceptable.

### Operating time and number of insertions of hysteroscope

The average operating time was significantly longer by using the electrosurgical snare: 11.7 minutes compared with 6.8 minutes in the hysteroscopic tissue removal group (p = 0.018). Also, the number of insertions of the hysteroscope was significantly higher in the snare group than in the tissue removal group,3.9 times versus 1.7 times, p < 0.001.

### Peri-operative and immediate postoperative complications

One serious adverse event occurred during the electrosurgical snare treatment. It was a uterine perforation, which was treated conservative but required prolonged hospital stay. No serious adverse events occurred in the hysteroscopic tissue removal group.

At two weeks of follow up 8/35 (23%) of the snare group versus 11/31 (36%) of the tissue removal group reported issues after the intervention. Respectively 7/35 (20%) and 7/31 (23%) of the women reported persistent vaginal blood loss. Other complaints were lower abdominal pain and fatigue. None of the complaints lasted longer than two weeks post-intervention. No severe postoperative complications were reported. No difference in post-operative complaints were found between women with a completely resected polyp and women with an incompletely resected polyp.

## Discussion

We compared the effectiveness of polyp removal of the electrosurgical polyp snare to the hysteroscopic tissue removal device. The electrosurgical polyp snare was found to be inferior to hysteroscopic tissue removal for complete resection of endometrial polyps. Secondary outcome measures showed no differences between the two techniques in pain scores, procedure acceptability and adverse events. The snare procedure had a significantly longer procedure time compared to the hysteroscopic tissue removal device.

Our results are in line with previously published studies. These studies show a completeness of polyp removal with the tissue removal device varying from 92%-100% compared to 77%- 95% removal with the snare (Smith et al., 2014; Tsuchiya et al., 2018; Hamerlynck et al., 2015). In our trial the hysteroscopic tissue removal device had a 95% completeness of resection, and the electrosurgical snare had a complete resection rate of 57%. An explanation of the difference of our results versus published data might be that the definition of completeness was handled differently across studies. More-over, completeness was judged by an independent researcher who was blinded for surgical technique.

We did not observe a significant difference in VAS scores between the two procedures. In the literature mean pain scores during the intervention favoured tissue removal (VAS 35.9 vs52.0 with electrosurgical resection) (Smith et al., 2014). In our study, no patient in the electrosurgical arm reported the intervention to be unacceptable and 9.5% of the women scored the hysteroscopic tissue removal device as unacceptable. This is different from published findings as poor pain tolerance is observed in 11% of the women undergoing electrosurgical resection and in 8% of the women that underwent resection with the hysteroscopic tissue removal device (Pampalona et al., 2015). In previous studies, the number of insertions of the hysteroscope was 2-8 times higher than with of the electrosurgical snare compared with the use of the hysteroscopic tissue removal device (Hamerlynck et al., 2015; Tsuchiya et al., 2018). In our study, the number of insertions of the hysteroscope was higher with the electrosurgical polyp snare at 3.9 insertions vs 1.7 with the hysteroscopic tissue removal device. The increase in insertion rate of the hysteroscope in the electrosurgical groups could explain the difference in tolerance scores.

The electrosurgical snare had a significantly longer operating time of 11.7 minutes compared to 6.8 minutes for the hysteroscopic tissue removal device. The published average operating time varies from 6 to 17 minutes for the electrosurgical snare and from 4 to 10 minutes for the hysteroscopic tissue removal device (Smith et al., 2014; Hamerlynck et al., 2015; Van Dongen et al., 2008). This agrees with our findings. However, these numbers do not consider the preparation time of the equipment. Although the hysteroscopic tissue removal device is quick to use, it takes significantly more time to prepare the instrument for use compared to the electrosurgical snare.

Various complications have been reported with the use of an electrosurgical polyp snare, such as uterine perforation and cervical damage due to repeated insertion of the device (Noventa et al., 2015). Studies that reviewed electrosurgical snares found that major complications were present in 0-7.5% of the interventions (Timmermans and Veersema, 2005; Hamerlynck et al., 2015; Tsuchiya et al., 2018; Van Dongen et al., 2008). Our findings reflect a similar complication with the electrosurgical snare with a 3% complication rate (n=1), due to a uterine perforation. The published intraoperative complication rate with the hysteroscopic tissue removal device is 1.6% in polypectomies (Smith et al., 2014; Pampalona et al., 2015; Van Dongen et al., 2008). No severe complications occurred in our study using the hysteroscopic tissue removal device.

At the 2 week follow up check, 20% of the patients in the snare group and 23% in the tissue removal device group reported persistent vaginal blood loss. This could not be attributed to an incomplete resection, as 19% of patients with incomplete resection experienced vaginal blood loss and 22% with a completely resected polyp also experienced vaginal bleeding. According to the literature the incidence of recurrent abnormal uterine bleeding is unaffected by the technique used from the intervention up until four years after the intervention (AlHili et al., 2013). Eventually nearly 60% of the women require further treatment for persistence or recurrence of abnormal uterine bleeding four years after hysteroscopic polypectomy (Henriquez et al., 2007).

Complete polyp removal is important as incomplete removal of the stalk and base of the polyp enhances the risk of polyp recurrence and thereby, occurrence of endometrial cancer (Yang et al., 2015). Postoperative recurrence rates vary from 2.5% to 43.6%, depending on the follow-up duration and nature of the polyp (Paradisi et al., 2014; AlHili et al., 2013). Longer duration of follow-up increases the risk of polyp recurrence. The type of polyp, either pedunculated or sessile, does not play a role in the recurrence (Yang et al., 2015). Also, the risk of recurrence is independent of the presence of abnormal uterine bleeding (Paradisi et al., 2014). Recurrence of endometrial polyps tends to be lower if women underwent tissue removal instead of hysteroscopic snare resection (AlHili et al., 2013). It is known that 4-6% of removed polyps are premalignant or malignant (Lee et al., 2010; Van Hanegem et al., 2017).

Histopathological examination of the resected polyps showed atypia in one polyp and malignancy in one polyp, which comes down to a total of 3% non-benign findings. This is slightly less than the known literature.

## Strengths and limitations

This trial is the first that provides an overview of the effectiveness of the electrosurgical polyp snare compared to the hysteroscopic tissue removal device. Previous studies merely focused on technical components such as ease of use, complications and/or surgical time. Furthermore, previous studies did not have a second and/or third independent investigator to score the resection as either ‘complete’ or ‘incomplete’. This strengthens the results of our trial by minimising bias.

This trial however has some limitations. First, the study was ceased prematurely because the polyp snare was withdrawn from the market due to reported complications and this meant that the calculated power could not be reached. Fortunately, we did not see any severe adverse events in our study due to snare complications. Second, the hysteroscopic images of 22 patients were accidentally deleted due to a technical fault in the computer system, so these patients could not be included in the analysis of the primary outcome.

Third, this study had a relatively short follow-up period of 2 weeks, making it unable to investigate the recurrence of polyps or development of (pre) malignancies. Nevertheless, this does not affect the primary outcome of this study.

## Conclusion

The electrosurgical polyp snare is inferior compared to hysteroscopic tissue removal for complete endometrial polyp resection. The use of the electrosurgical snare results in a significant longer operation time, more insertions of the hysteroscope and a slightly higher complication rate. Because of the higher rate of completeness of resection, the overall safety and acceptability of the hysteroscopic tissue removal device, the implementation of this tool in both outpatient and in-patient hysteroscopic polypectomy is recommended over the electrosurgical polyp snare.
